# Hydrogenolysis of 5-Hydroxymethylfurfural to 2,5-Dimethylfuran Over a Modified CoAl-Hydrotalcite Catalyst

**DOI:** 10.3389/fchem.2022.907649

**Published:** 2022-04-28

**Authors:** Jing Xia, De Gao, Feng Han, Ruifu Lv, Geoffrey I. N. Waterhouse, Yan Li

**Affiliations:** ^1^ College of Chemistry and Material Science, Shandong Agricultural University, Taian, China; ^2^ School of Chemical Science, The University of Auckland Private Bag, Auckland, New Zealand

**Keywords:** 5-hydroxymethylfurfural, 2,5-dimethylfuran, hydrogenolysis, hydrotalcite, metal oxide

## Abstract

The catalytic hydrogenolysis of 5-hydroxymethylfurfural (HMF) to 2,5-dimethylfuran (DMF) is a promising route towards sustainable liquid fuels with a high energy density. Herein, a novel CuCoNiAl-containing mixed metal oxide catalyst (CuCoNiAl-MMO) was prepared by calcination a layered double hydroxide (LDH) precursor in N_2_ at 500 °C, then applied for the catalytic hydrogenolysis of HMF to DMF. The effects of reaction time, reaction temperature and hydrogen pressure on DMF selectivity were investigated. Under relatively mild reaction conditions (180°C, 1.0 MPa H_2_, 6.0 h), CuCoNiAl-MMO showed both a high initial activity and selectivity for hydrogenolysis of HMF to DMF, with HMF conversion rate of 99.8% and DMF selectivity of 95.3%. Catalysts characterization studies using scanning electron microscopy (SEM), transmission electron microscopy (TEM), X-ray diffraction (XRD) and X-ray photoelectron spectroscopy (XPS) revealed the presence of various metal oxides and metallic copper on the surface of the CuCoNiAl-MMO catalyst, with the presence of mixed metal-oxide-supported metallic Cu nanoparticles being responsible good hydrogenolysis activity of the catalyst for selective DMF synthesis.

## 1 Introduction

**FIGURE 1:**
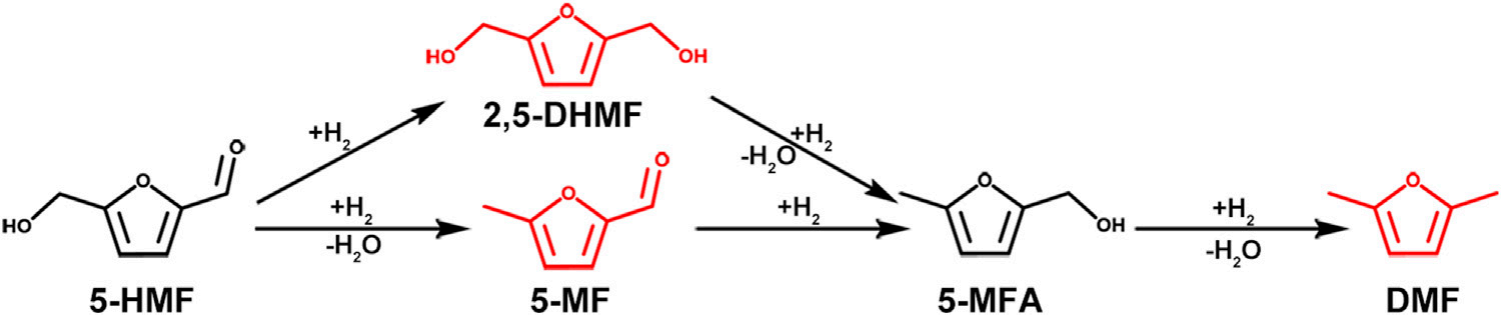
Two reaction pathways for the hydrogenolysis of HMF to DMF.

Climate change caused by anthropogenic greenhouse gas emissions is a major environmental concern (Bond, Doherty et al., 2013). Most of these emissions are in the form of CO_2_ from the burning of fossil fuels for energy. Therefore, in order to reduce the harm to the environment and reduce the dependence on fossil fuel energy, the search for low-cost, safe, environmentally friendly, sustainable development of new energy has become a growing concern of people. Amongst fuels which can be produced sustainably, biomass derived products represent an excellent choice (Nakagawa et al., 2013; Zhang and Deng, 2015). Biomass can be converted into chemical energy and stored by photosynthesis, which is then converted into valuable chemicals and fuels by appropriate treatment methods. 5-Hydroxymethylfurfural (HMF), as a biomass platform compound readily obtained from sucrose and fructose, can be catalytically converted into a range of useful products, including 2,5-bis(hydroxymethyl)furan (DHMF), 1,6-hexanediol (HDO), 2,5-dimethyltetrahydrofuran (DMTHF), and 2,5-dimethylfuran (DMF) (Wang Q. et al., 2019). In these compounds, DMF is a liquid biofuel with high octane number, water insolubility, and high energy density. These characteristics are similar to many existing transportation fuels, making DMF an ideal and promising transportation liquid biofuel (Viar et al., 2019). The aldehyde group and hydroxymethyl group on the furan ring of 5-HMF are easy to react in the presence of catalyst and hydrogen donor, and hydrodehydration becomes DMF. In general, there are two possible reaction pathways for conversion of HMF into DMF, as shown in Figure 1. In Pathway 1, the aldehyde group on HMF is first hydrogenated to form DHMF, followed by hydrohydrolysis of the hydroxyl group on the furan ring to form 5-methylfurfuryl alcohol (5-MFA), an important reaction intermediate in the synthesis of DMF. In Pathway 2, the hydroxyl group in HMF hydrolyzes and dehydrates to generate 5-methylfurfural (5-MF), then the aldehyde group on 5-MF is hydrogenated to generate 5-MFA, after which the hydroxyl group on 5-MFA is further hydrolyzed to DMF.

To date, heterogeneous noble metal-based [such as Pt (Luo et al., 2016; Shi et al., 2016), Ru (Feng et al., 2020; Wang et al., 2020) and Pd (Nakagawa and Tomishige, 2010; Zhang J. et al., 2019)] catalysts offer the best all-round performance in the field of HMF hydrogenation to DMF. Luo et al. prepared a carbon-supported Pt_3_Ni alloy nanoparticle catalyst, which efficiently converted HMF to DMF (98% yield) at 3.3 MPa H_2_ and 160–200°C (Luo et al., 2016). Zu et al. prepared a Ru/Co_3_O_4_ catalyst by simple coprecipitation method. Under low reaction temperature and H_2_ pressure conditions (130°C, 0.7 MPa), the yield of DMF reached 93.4% (Zu et al., 2014). Mhadmhan et al. prepared a Cu-Pd bimetallic catalyst on reduced graphene oxide (RGO), which offered good performance for the hydrogenation of HMF to DMF with 2-propanol as a hydrogen donor (96% HMF conversion and 95% DMF yield) (Mhadmhan et al., 2019). However, the high cost of these noble metal-based based catalysts are obstacles for the large scale conversion of HMF to DMF, motivating the search for low-cost catalysts based on non-noble metals. Dinesh et al. synthesized a Ni-Cu/HT catalyst by the deposition precipitation method, achieving a DMF yield of 83% at 85°C, 0.6 MPa H_2_ pressure and 100 mg catalyst load (Gupta et al., 2020). Chen et al. prepared carbon-coated Cu-Co bimetallic nanoparticles (Cu-Co@Carbon) by heat-treating a bimetallic oxide precursor. The Cu-Co@Carbon catalyst afforded a DMF yield of 99.4% at 180°C and a H_2_ pressure of 5 MPa over 8 h in a batch reactor, which was superior to supported precious metal catalysts (Chen et al., 2017). Yang et al. prepared a 2%Ni-20%Co/C catalyst, which was capable of selectively producing DMF in a high yield (95%) from biomass-derived HMF under mild conditions (130°C, 1 MPa H_2_) (Yang et al., 2016). In these catalysts, synergies between the supported metals and the acid-base surface sites on the support promote the activation of hydrogen, thereby resulting in enhanced catalyst activity for the hydrogenolysis of HMF to DMF (Yang et al., 2016; Gupta et al., 2020).

Layered double hydroxides (LDHs), also known as hydrotalcite-like compounds (HTlcs), are a type of 2D layered material with general formula [(M^2+^)_1−x_ (M^3+^)_x_ (OH)_2_]^x+^(A^m−^_x/m_)·nH_2_O]. The LDH structure consists of positively charge sheets containing divalent and trivalent metal cations ions octahedrally coordinated by oxygen, with the interlayer region containing charge-balancing anions and water molecules. Calcination of LDH materials in air or an inert atmosphere above certain temperatures results in collapse of the 2D layered structure and the formation composite oxides (MMO) *via* topological transformation processes. Depending on the temperature and calcination environment, sometimes supported metal nanoparticle catalysts can be obtained. The LDH-derived catalyst obtained via these approaches typically possess the advantages of large specific surface area, high thermal stability and excellent dispersion of the metal and metal oxide phases (Xu et al., 2011; Mishra et al., 2018). Owing to the wide range of cations that can be accommodated in the LDH structure (e.g. divalent cations include Ca^2+^, Mg^2+^, Fe^2+^, Co^2+^, Mn^2+^, Ni^2+^, Cu^2+^, or Zn^2+^ and trivalent cations include Al^3+^, Fe^3+^, Co^3+^, and Ni^3+^), it is possible to make a diverse range of catalysts with different surface properties via this strategy. Zhang et al. used a hydrotalcite precursor to synthesize an inexpensive copper-based catalyst for the selective transfer hydrogenation of biomass-based HMF with methanol as both a solvent and hydrogen donor (Zhang and Chen, 2017). Ma et al. prepared a NiCoTi-8 catalyst by heat treatment of a NiCoTi-LDH precursor, with the catalyst offering good performance for the hydrogenolysis of HMF to DMF (Ma et al., 2020). Zhang et al. prepared a CuZnCoO_x_ ternary catalyst, which converted HMF to DMF (yield 99%) in the presence of ethanol as a hydrogen donor (Zhang Z. et al., 2019). The intercalation of the small anionic metal complexes in the interlayer region of LDH provides a further approach for modifying the chemical composition and physical properties (e.g. specific surface area and surface acidity/basicity) of the LDH-derived MMO catalysts (Ma et al., 2011; Zhang J. et al., 2019; Qin et al., 2020). Tailoring the surface composition and surface properties of the catalysts is therefore a good approach for realizing a high HMF conversion and selective DMF production.

In this study, a four-metal hydrotalcite CuCoNiAl-LDH precursor was prepared by a one-pot hydrothermal method. After calcination at 500°C in a nitrogen environment, CuCoNiAl-MMO was obtained, which was then applied HMF hydrogenation to generate DMF. CuCoNiAl-MMO afforded a HMF conversion of 99.8% rate and DMF yield of 95.3% under mild conditions (180°C, 1.0 MPa H_2_), and could be recycled three times whilst maintaining stability. Characterization studies verified that the formation of MMO-supported zero-valent Cu and Co nanoparticles accounted for the excellent performance of the CuCoNiAl-MMO catalyst in the hydrogenolysis of HMF.

## 2 Materials and Methods

### 2.1 Materials

Copper nitrate trihydrate (Cu(NO_3_)_2_·3H_2_O, ≥99%) was purchased from Aladdin Chemical Trading Co. Ltd. Cobalt (II) nitrate hexahydrate (Co(NO_3_)_2_·6H_2_O, ≥98%), nickel (II) chloride hexahydrate (NiCl_2_·6H_2_O, ≥98%), and 5-hydroxymethyl furfural (C_6_H_6_O_3_, ≥98%) were purchased from Shanghai D&B Biological Science and Technology Co. Ltd. Aluminum nitrate nonahydrate (Al(NO_3_)_3_·9H_2_O, >97%) was purchased from BASF Chemical Trading Co. Ltd. (TianJin, China). Urea (CO(NH_2_)_2_, ≥99.0%), ethanol absolute (CH_3_CH_2_OH, ≥99.7%), and tetrahydrofuran (C_4_H_8_O, ≥99.0%) were purchased from Tianjin Kaitong Chemical Reagent Co. Ltd. The experimental water was deionized water. The hydrogen used in the experiment was 99.9% pure.

### 2.2 Preparation of CuCoNiAl-MMO

First, the catalyst precursor CuCoNiAl-LDH was prepared using a hydrothermal method. Briefly, 0.01 mol Cu(NO_3_)_2_·3H_2_O, 0.01 mol Co(NO_3_)_2_·6H_2_O, 0.01 mol NiCl_2_·6H_2_O, 0.01 mol Al(NO_3_)_3_·9H_2_O and 0.15 mol of urea were dissolved in 100 ml of deionized water. The molar ratio of Cu/Co/Ni/Al was 1:1:1:1. The resulting solution was then stirred at room temperature for 1 h. Next, the solution was transferred to a 100 ml autoclave and hydrothermal reacted in an oven at 120°C for 6 h. After the reaction device was cooled to room temperature, the solid product was collected, washed with deionized water to pH = 7, and dried at 90°C for 10 h. The product obtained is denoted herein as CuCoNiAl-LDH. CuCoNiAl-MMO was obtained by calcination CuCoNiAl-LDH in a tube furnace at 500°C under a nitrogen atmosphere for 5 h. For reference purposes, several other LDH precursors (CoAl-LDH and CuCoAl-LDH) were also prepared, which yielded CoAl-MMO and CuCoAl-MMO, respectively, after calcination at 500°C in nitrogen environment.

### 2.3 Hydrogenation Tests

The catalytic hydrogenation experiments of HMF were carried out in a 50 ml autoclave. In a typical experiment, the reactor was charged with 0.25 g of HMF, 0.1 g of catalyst, and 10 ml of tetrahydrofuran (THF). To remove other gases, the reactor was purged three times with H2 before the reaction. Then, the reactor was charged with H_2_ (1.0 MPa). Reactions were carried out at 180°C for 6 h with stirring at 560 rpm. After the reaction, the reaction solution was collected, and the supernatant was collected by centrifugation. Biphenyl (0.1 g) was then added to the supernatant liquid as an internal standard. Finally, the liquid samples were analyzed on a Shimadzu GC-2010 gas chromatograph equipped with a AE-SE-54 column and a flame ionization detector (FID). The HMF conversion and product selectivities were calculated with reference to the internal standard.

### 2.4 Catalyst Recycling Tests

After the HMF hydrogentation test, the used solid catalyst was collected by centrifugation, washed with ethanol three times, then dried at 80°C in the oven. Finally, the catalyst was heated at 500°C for 5 h in a tube furnace before reuse.

### 2.5 Catalyst Characterization

SEM analysis was performed on NoVaTM Nano SEM 430 scanning electron microscope (FEI, United States) with electron acceleration voltage of 20 kV. High-resolution transmission electron microscopy (HRTEM) images were acquired on a FEI Tecnai G20 (United States) instrument with an accelerating voltage of 300 kV. The XRD patterns were obtained by Japanese Rigaku Smartlab SE XRD diffractometer, with Cu Kα source (λ = 0.154 nm, 40 kV) as the light source, and the scanning speed of data acquisition was 2°/min. TGA analysis was performed in air using a thermogravimetric analyzer (TA SDT Q600). The N_2_ flow rate was 100 ml/min and the heating rate was 10°C/min. FT-IR spectra were in the wavelength range of 4,000–400/cm and were collected on a Thermo Scientific Nicolet 380 spectrometer (United States). XPS maps were obtained on the K-alpha XPS system (Thermo Fisher Scientific, United States) equipped with a monochrome Al Kα source (hν = 1,486.6 eV). Binding energy scale calibration was performed using C 1s (284.8 eV). N_2_ adsorption and desorption isotherms at 77 K were measured on a Tristar 3,000 instrument (Micromeritics Instrument Corp). Samples were degassed at 373 K for 12 h before data acquisition. BET and BJH methods were used to calculate the surface area and pore volume of samples, respectively. Temperature-programmed desorption experiments of NH_3_ (NH_3_-TPD) were performed on an AutoChem II 2920 V5.02 instrument (Micromeritics instrument Corp). In the NH_3_-TPD experiment, 100 mg of the catalyst was pretreated under argon atmosphere at 300°C for 2 h to remove the surface adsorbed species. The catalyst was then cooled to 50°C and exposed to a 2000 ppm NH_3_/Ar atmosphere for 2 h to achieve NH_3_ adsorption saturation. Then the catalyst was reacted in 50 ml/min argon solution for 1 h, and finally the catalyst was heated from 50 to 800°C at a heating rate of 10°C/min. The thermal conductivity detector (TCD) detected the change of NH_3_ with temperature. Pyridine infrared (Py-IR) spectra were obtained by testing on a PE Frontier FT-IR spectrometer (PerkinElmer). The sample was vacuum-degassed at 350°C for 2 h, followed by adsorption of pyridine vapor (saturated vapor pressure) at room temperature for 0.5 h. For H_2_-TPR analysis, the catalyst was heated at 300°C for 2 h under a He atmosphere, then cooled to 50°C and kept at a flow of He (50 ml/min) for 1 h. Next, a mixed gas of 5% H_2_/He (50 ml/min) was introduced and the catalyst was heated from 50 to 800°C at a rate of 10°C/min. Elemental analysis data were collected by atomic emission spectrometer (ICP-AES).

## 3 Results and Discussion

### 3.1 Catalyst Characterization

#### 3.1.1 SEM

**FIGURE 2:**
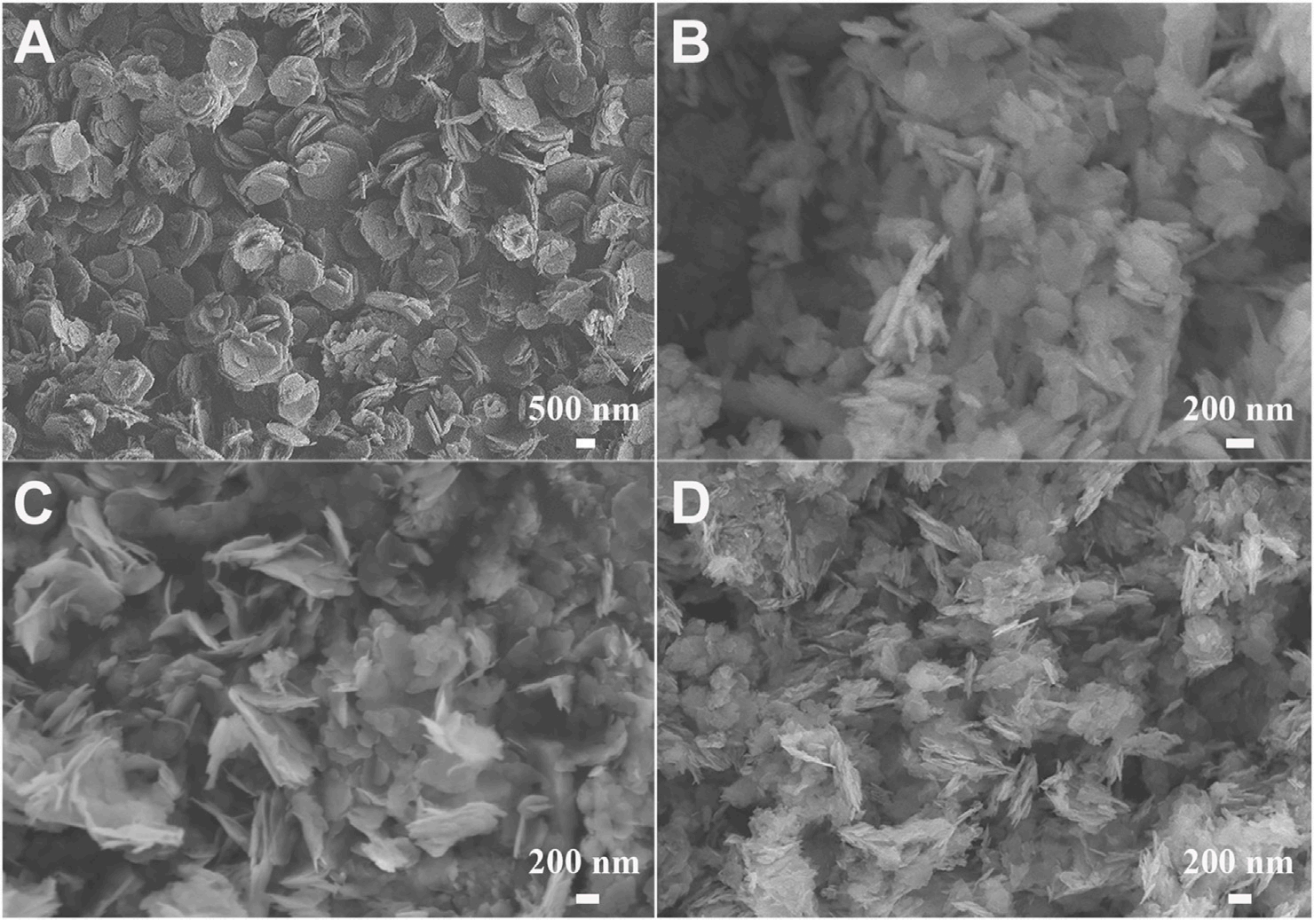
SEM images of **(A)** CoAl-LDH, **(B)** CuCoAl-LDH, **(C)** CuCoNiAl-LDH, and **(D)** CuCoNi**A**l-MMO.

The morphologies of the CoAl-LDH, CuCoAl-LDH and CuCoNiAl-LDH precursors were first examined by SEM (Figure 2). The CoAl-LDH sample consisted of uniformly sized hexagonal platelets that were stacked on top of each other (Figure 2A). This structure is typical for LDH materials. The CuCoAl-LDH sample also contained stacked platelets, but the platelet size and shape were less uniform (Figure 2B). The morphology of CuCoNiAl-LDH was similar to that of CuCoAl-LDH (Figure 2C). CuCoNiAl-LDH was calcined under N_2_ at 500°C for 5 h to obtain CuCoNiAl-MMO (Figure 2D), which retained some of the stacked platelet structure of the LDH precursor.

#### 3.1.2 TEM

**FIGURE 3:**
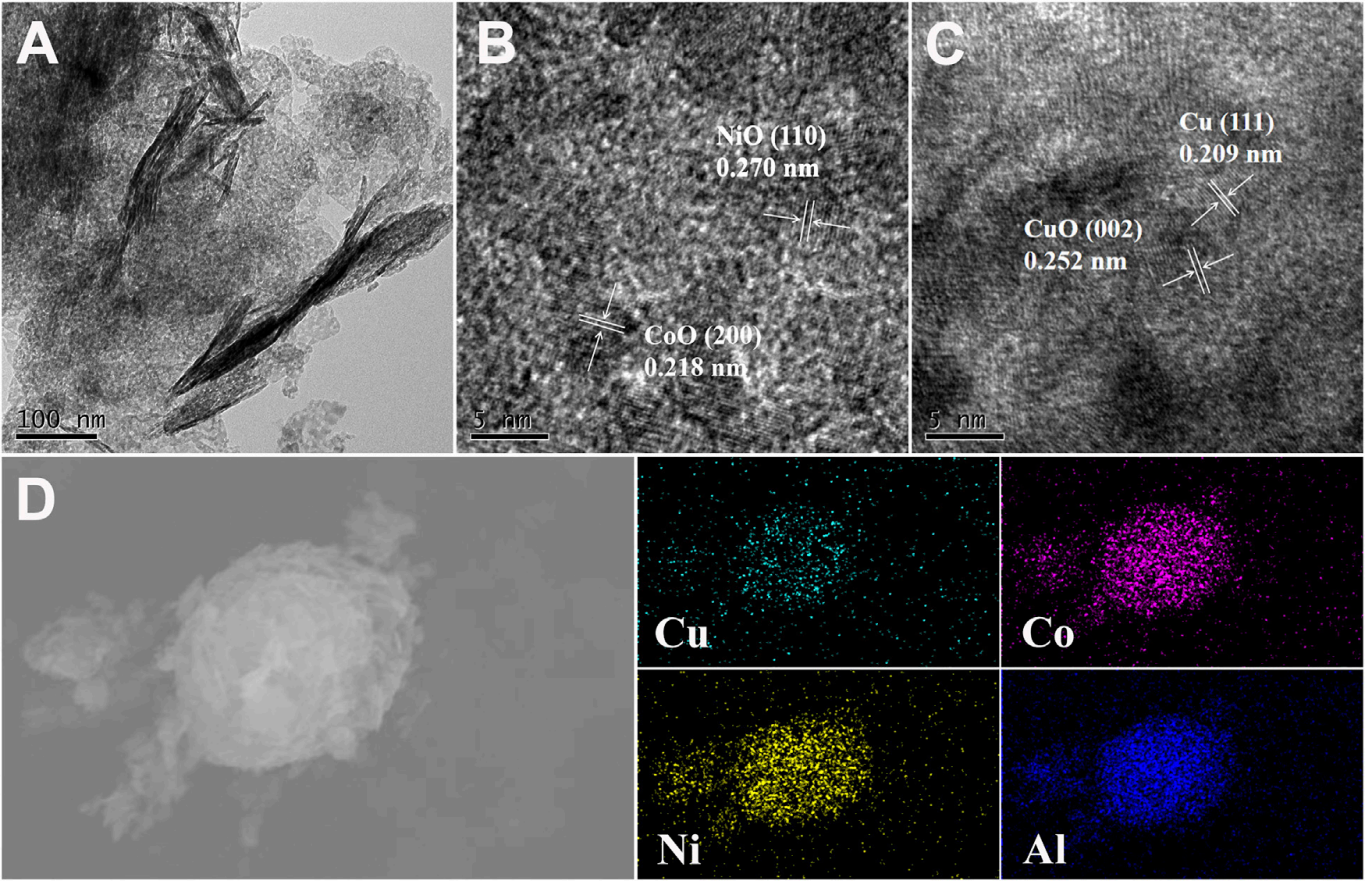
**(A)** TEM and **(B)**, **(C)** HRTEM images of CuCoNiAl-MMO, **(D)** SEM-EDS image and corresponding element maps for CuCoNiAl-MMO.

In order to better understand the structure and element composition of the CuCoNiAl-MMO catalyst, TEM and HRTEM analyses were performed. Figure 3A shows that CuCoNiAl-MMO retained some of the layered structure of the LDH precursor. HRTEM images of CuCoNiAl-MMO (Figures 3B,C) provided more detailed information about the components present in the sample. In Figure 3B, nanoparticles with lattice fringes of 0.218 and 0.270 nm are observed, corresponding to CoO (200) and NiO (110) planes, respectively (Zhu et al., 2018; Li et al., 2019). In Figure 3C, nanoparticles with lattice fringes of 0.209 and 0.252 nm are observed, which can readily be assigned to Cu (111) and CuO (002) planes, respectively (Gao et al., 2018; Luo et al., 2020). No obvious latticle fringes associated with an Al-containing phase were seen, suggesting that alumina was likely present in an amorphous phase (i.e. an amorphous alumina). Next, the element distribution in CuCoNiAl-MMO was probed by EDS element mapping analysis. As shown in Figure 3D, Cu, Co, Ni and Al were are evenly distributed throughout the catalyst, consistent with expectations for LDH-derived catalysts. Results suggests that the four metals (in their respective metallic an oxide forms) were in intimate contact and thus could potentially act synergistically to boost the catalytic activity of CuCoNiAl-MMO for HMF hydrogenation.

#### 3.1.3 XRD

**FIGURE 4:**
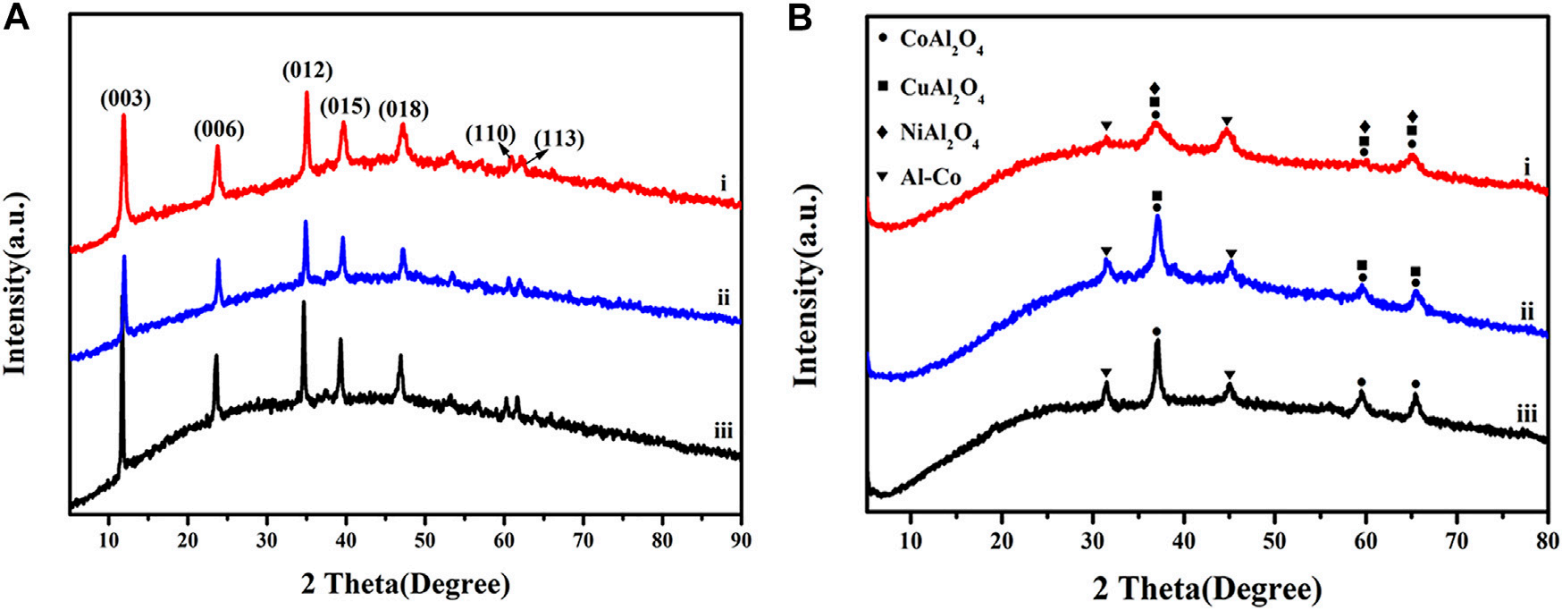
XRD patterns for **(A)** CuCoNiAl-LDH **(i)**, CuCoAl-LDH **(ii)** and CoAl-LDH **(iii)**, and **(B)** CuCoNiAl-MMO **(I)**, CuCoAl-MMO **(ii)** and CoAl-MMO **(iii)**.

Figure 4A shows XRD patterns for the LDH precursors. All show characteristic sets of (003), (006) and (012) reflections at 11.8, 23.7 and 34.8° typical for stacked LDH materials. After calcination at 500°C in N_2_, the characteristic reflections disappeared due to collapse of the LDH interlayers, and were instead replaced by peaks associated mainly with oxide phases, as shown in Figure 4B. The XRD patterns for CuCoNiAl-MMO, CuCoAl-MMO, and CoAl-MMO were similar, showing peaks at similar positions and peaks with the same relative intensities, consistent with the formation of spinel-like MAl_2_O_4_ oxides (Gao et al., 2018). The XRD pattern of CoAl-MMO contained peaks at 37.1, 59.5 and 65.5°, corresponding to the (311), (511) and (440) phases of CoAl_2_O_4_ (PDF#-38–0,814), respectively. In addition, further peaks were observed around 31.4° and 45.0°, typical for the (100) and (110) reflections, respectively, an Al-Co alloy (PDF#-29–0,021). The XRD patterns of CuCoAl-MMO, showed peaks at 37.1, 59.5 and 65.5°, corresponding to the (220), (311) and (440) crystal planes, respectively, of CuAl_2_O_4_ (PDF#-33–0,448). The presence of the Al-Co alloy was also evident by peaks at 31.4° and 45.0°. For CuCoNiAl-MMO, in addition to the CoAl2O4 and CuAl2O4 phases, peaks at 37.1, 59.5 and 65.5° might be due to the (311), (511) and (440) reflections of NiAl_2_O_4_ (PDF#-10–0,339). These overlap with peaks for CuAl_2_O_4_. By HRTEM, lattice fringes associated with Cu metal, NiO, CuO and CoO were seen. These phases were not detected by XRD, suggesting that were surface phases and present in relatively low abundance relative to the MAl_2_O_4_ and Al-Co phases. Cu_2_AlO_4_, CoAl_2_O_4_, and NiAl_2_O_4_ belong to the spinel phase, which are typical metal-based oxides with high catalytic activity, and their tetrahedral and octahedral centers provide multiple sites to accommodate different metal cations (Dasgupta et al., 2016; O'Quinn et al., 2017; Qi et al., 2022). In addition, the spinel phase is thermally stable, which can make the structure of the catalyst more stable (Ma et al., 2011). The Al-containing component acts both as a carrier and as a physical spacer to suppress the aggregation of metal particles, thereby ensuring high dispersion (Gao et al., 2018).

#### 3.1.4 XPS

**FIGURE 5:**
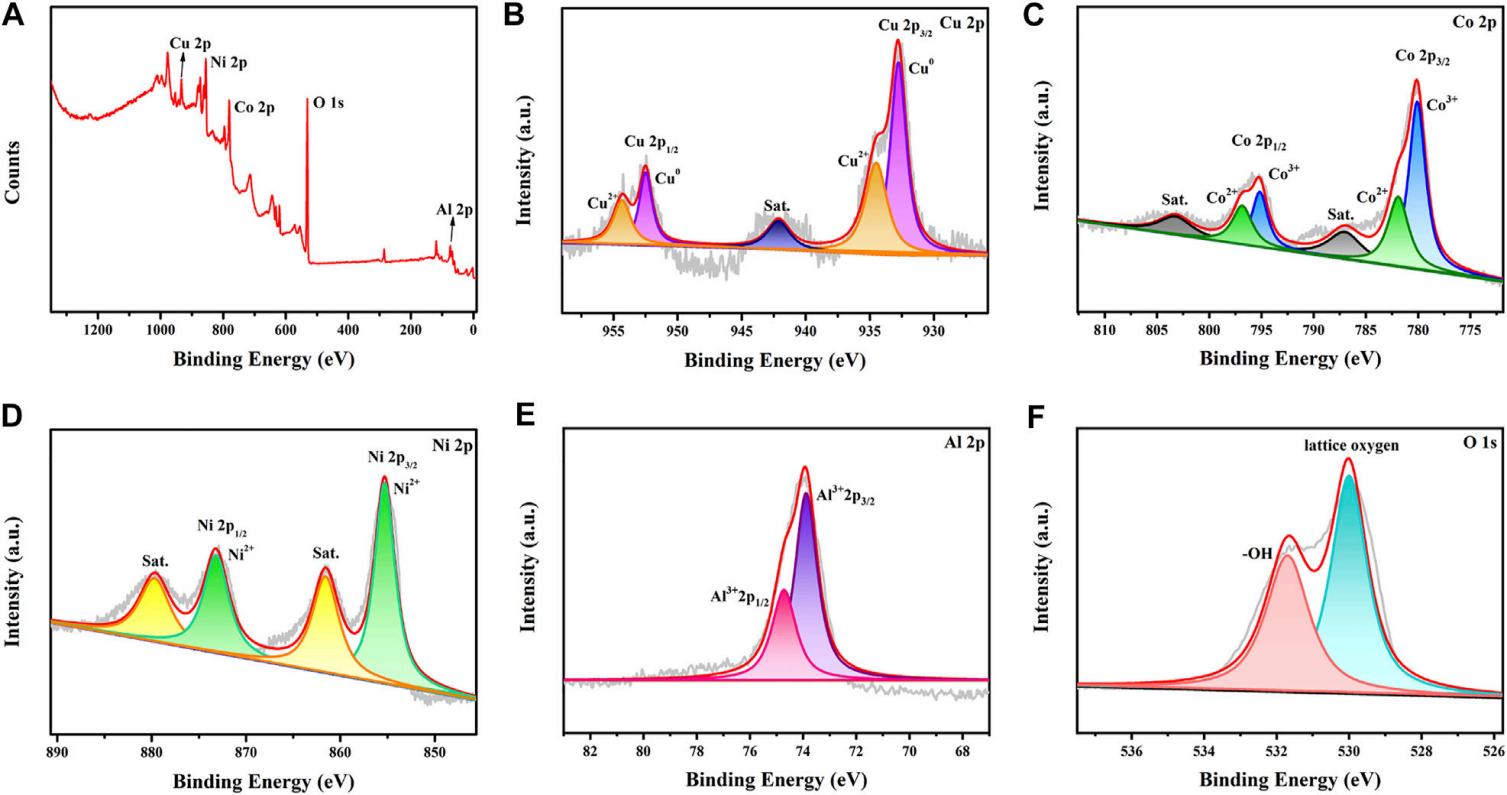
XPS spectra for CuCoNiAl-MMO samples: **(A)** survey spectrum; **(B)** Cu 2p spectrum; **(C)** Co 2p spectrum; **(D)** Ni 2p spectrum; **(E)** Al 2p spectrum and **(F)** O 1s spectrum.

The valence states of the metals in the CuCoNiAl-MMO catalyst were probed by XPS. The survey spectrum of CuCoNiAl-MMO is shown in Figure 5A, revealing the presence of Cu, Co, Ni, Al and O elements. The Cu 2p XPS spectrum showed three characteristic sets of peaks, each having a 2:1 area ration for the 2p_3/2_ and 2p_1/2_ peaks. The Cu 2p_3/2_; Cu 2p_1/2_ and satellite peaks at 932.6, 952.5 and 942.1 eV, respectively, indicate the presence of Cu species exists in the form of Cu^0^ (2p_3/2_ = 932.8 eV, 2p_1/2_ = 952.5 eV) and Cu^2+^ (2p_3/2_ = 934.5 eV, 2p_1/2_ = 954.3 eV) in CuO or Cu_2_AlO_4_ (Figure 5B) (Shang et al., 2020; Umasankar et al., 2020). The Co 2p XPS spectrum (Figure 5C) showed two distinct sets of peaks: Co^2+^ (2p_3/2_ = 781.9 eV, 2p_1/2_ = 796.8 eV) and Co^3+^ (2p_3/2_ = 780.3 eV, 2p_1/2_ = 795.2 eV). In addition, two Co 2p shake-up satellites were observed at 787.8 and 803.5 eV (Liao et al., 2020). Therefore, it can be concluded that cobalt exists mainly existed in the form of oxides in the near surface region of the sample (CoAl alloys seen by XRD may have been surface oxidized). The Ni 2p XPS spectrum (Figure 5D) showed peaks at 855.2 and 873.1 eV (2:1 area ratio), which can be easily assigned to the Ni 2p_3/2_ and Ni 2p_1/2_ peak, respectively of Ni(II) (Ma et al., 2020) in NiO or NiAl_2_O_4_. The presence of shake-up satellite peaks in the Ni 2p spectrum further confirmed the presence of Ni^2+^. The Al 2p spectrum (Figure 5E) can be deconvoluted into two Gauss-Lorentz sub-peaks at 73.8 and 74.7 eV (2:1 area ratio), which were assigned to an Al^3+^-containing oxide species (2p_3/2_ and 2p_1/2_, respectively) (Motamedi and Cadien, 2014). The O 1s (Figure 5F) XPS spectrum for CuCoNiAl-MMO contained peaks due to lattice oxygen in metal oxides (529.9 eV) and -OH (531.5 eV). The XPS data are basically consistent with the XRD characterization results.

#### 3.1.5 TG–DTA

**FIGURE 6:**
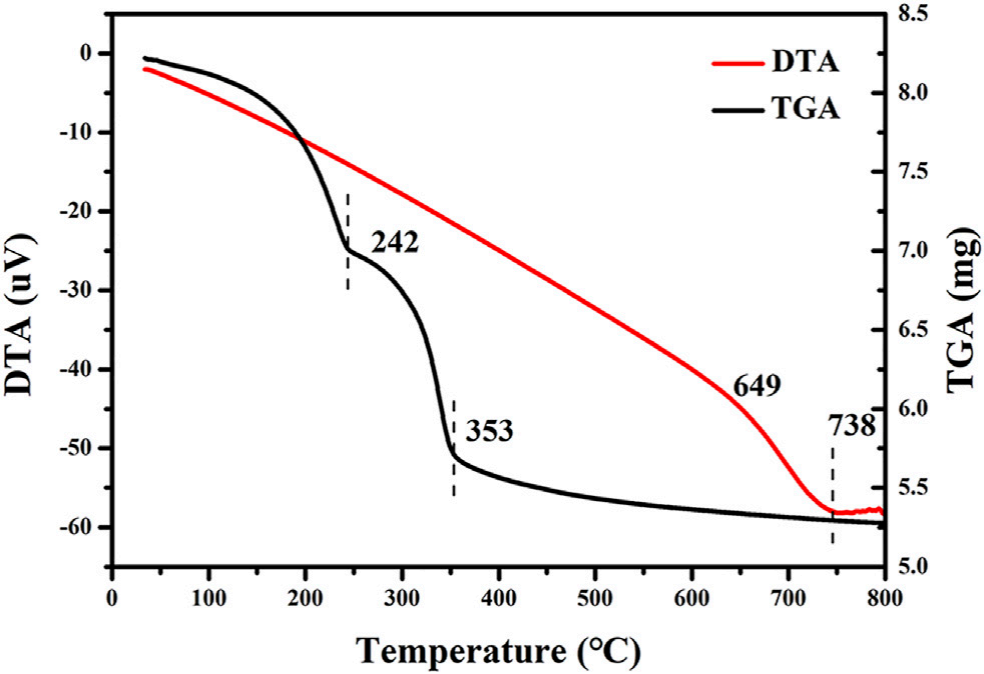
TG-DTA curves for CuCoNiAl-LDH in N_2_. The heating rate was 10°C/min.

Figure 6 shows the TG-DTA curve obtained by calcination CuCoNiAl-LDH in N_2_. A large mass loss was observed around 242°C, resulting from the removal of water from the LDH interlayer (Ardanuy and Velasco, 2011). Greater mass loss occurred between 242–353°C, which is associated with dehydroxylation of LDH flakes and decomposition of nitrate/carbonate anions in the interlayer (Zheng and Chen, 2017). Above 353°C, the product was stable against large mass losses. Based on the TG-DTA curves, a temperature of 500°C was selected for the preparation of the CuCoNiAl-MMO catalyst.

#### 3.1.6 FT-IR

**FIGURE 7:**
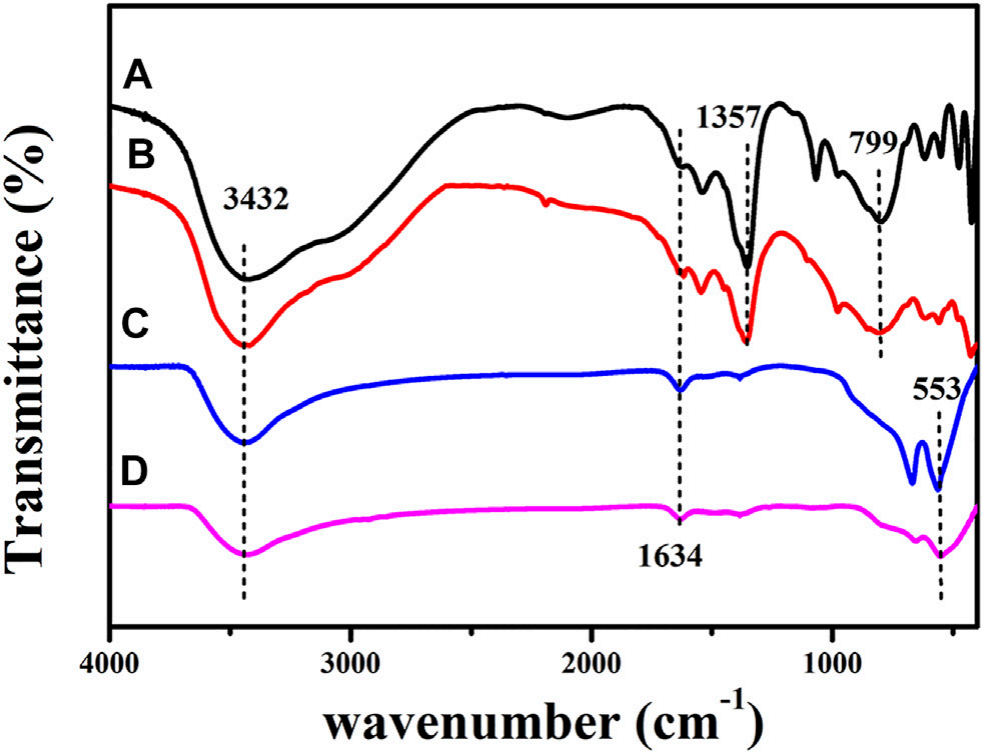
FT-IR spectra for CuCoAl-LDH **(A)**, CuCoNiAl-LDH **(B)**, CuCoAl-MMO **(C)** and CuCoNiAl-MMO **(D)**.

Figure 7 shows the FT-IR spectra for CuCoAl-LDH, CuCoNiAl-LDH, CuCoAl-MMO, and CuCoNiAl-MMO. The FT-IR spectra of CuCoAl-LDH and CuCoNiAl-LDH showed a broad absorption peak at 3,432 cm^−1^, due to O-H stretching vibrations of the hydroxyl groups and water in the LDH interlayer region. The bending vibration of the interlayer water molecules occurs at 1,634 cm^−1^. The peaks at 799 cm^−1^ and 1,357 cm^−1^ belong to the stretching of CO_3_^2−^ (Zheng and Chen, 2017) ions in the LDH interlayer. In an aqueous solution containing dissolved CO_2_, the interlayer nitrate of LDHs are easily exchanged for carbonate (Auxilio et al., 2008). After calcination at 500°C, the sharp absorption peak representing CO_3_^2−^ disappeared completely. New features appeared below 800 cm^−1^, which are related to M-O stretching vibrations (Li et al., 2017). This further proves that oxide phases were the main product of LDH calcination in N_2_.

#### 3.1.7 Specific Surface Area

The specific surface area and porosity of CuCoNiAl-LDH and CuCoNiAl-MMO were determined by N_2_ physisorption at 77 K. Table 1 shows the specific surface area and pore size distribution data of CuCoNiAl-LDH and CuCoNiAl-MMO calculated by Brunauer Emmett Teller (BET) and Barrett Joyner Halenda (BJH) methods, respectively. The BET specific surface area of CuCoNiAl-MMO was 2.5 times higher than that of the LDH precursor. The BJH cumulative pore volume of CuCoNiAl-MMO was 1.74 times that of CuCoNiAl-LDH, consistent with the former containing smaller mesopores. Results show that the collapse of the laminate structure in CuCoNiAl-LDH with calcination to 500°C resulted in a solid product with a high surface area and abundant pores (Ma et al., 2011), as is evident in the SEM image of CuCoNiAl-MMO in Figure 2D.

**TABLE 1:** Summary of N_2_ physisorption-data for CuCoNiAl-LDH and CuCoNiAl-MMO.

Catalyst	BET surface area (m^2^/g)	BJH surface area (m^2^/g)	Pore volume (cm^3^/g)	Pore diameter (Å
CuCoNiAl-LDH	59.2	66.4	0.48	286.7
CuCoNiAl-MMO	148.6	168.2	0.83	197.1

#### 3.1.8 H_2_-TPR

**FIGURE 8:**
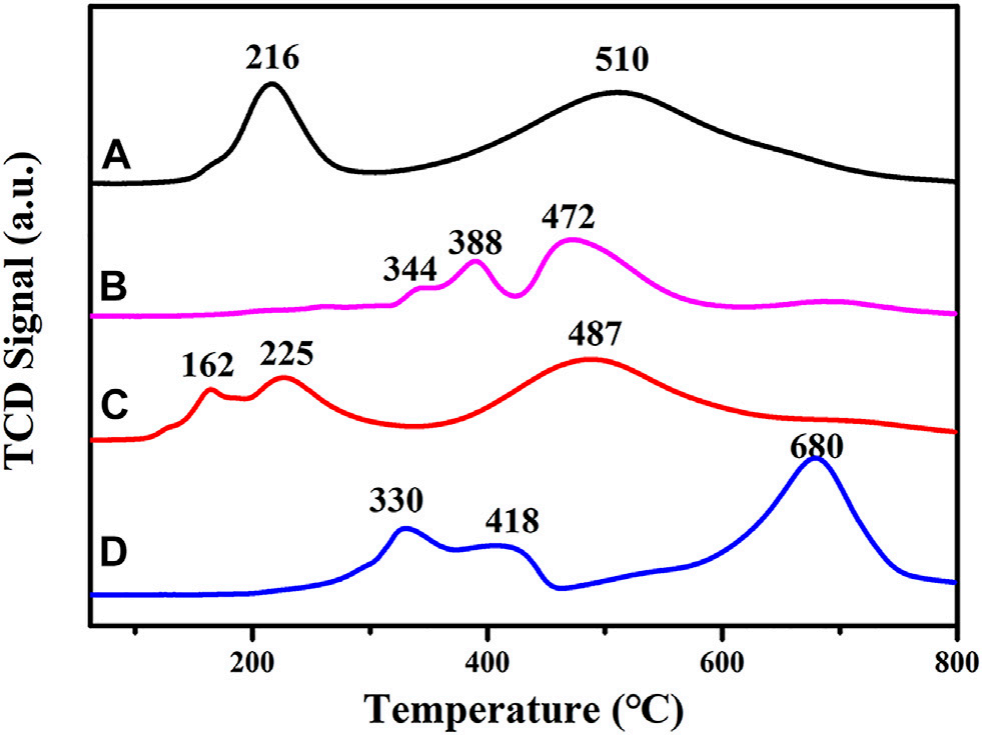
H_2_-TPR curves for **(A)** CoAl-MMO, **(B)** CuCoAl-MMO, **(C)** CuCoNiAl-MMO, and **(D)** CuCoNiAl-MMO-3.

H_2_-TPR spectra for the four catalysts, CoAl-MMO, CuCoAl-MMO, CuCoNiAl-MMO and CuCoNiAl-MMO-3 (the aged catalyst after three cycles of HMF hydrogenation) are shown in Figure 8. For the CoAl-MMO sample (curve a), two H_2_ consumption peaks are observed at 216 and 510°C. The first peak at 216°C is due to the reduction of Co_3_O_4_ to CoO, and the second peak at 510°C is due to the reduction of CoO to metallic cobalt (Zu et al., 2014). For CuCoAl-MMO, three reduction peaks are found. The peaks are associated with the two step reduction of Cu^2+^ to Cu^+^ to Cu (Gao et al., 2018) and the higher temperature peak with the reduction of Co^2+^ (i.e., partial reduction of CuAl_2_O_4_/Co_2_AlO_4_ (Koso et al., 2009; Shi et al., 2016)). The lowering of the reduction temperature for the Co^2+^ to Co metal transition in the presence of Cu^2+^ may be due to the strong interaction between Cu^2+^ and the Co/Al oxide matrix (Wang X. et al., 2019). Thee reduction peaks were seen in H_2_-TPR spectrum of CuCoNiAl-MMO (curve c) at 162, 225 and 487°C. The peak at 162°C is due to the reduction of CuO to Cu^+^, the peak at 225°C to the reduction of Co^3+^ to Co^2+^ (as CoO), and the peak at 487°C to the reduction of CoO to cobalt metal. Based on the results, in can be concluded that cobalt in the samples is closely associated with copper and nickel. These interactions promote the reduction of Co and were expected to modify the performance of the catalyst (Gupta et al., 2020; Umasankar et al., 2020). The used CuCoNiAl-MMO-3 catalyst showed TPR peaks at 330, 418, and 680°C. These peaks are discussed below.

### 3.2 Catalytic Performance Analysis

#### 3.2.1 Catalyst Performance Tests

Following the catalyst characterization studies, the catalytic performance of the three catalysts CoAl-MMO, CoNiAl-MMO, CuCoAl-MMO and CuCoNiAl-MMO for the conversion of HMF to DMF was studied. Experiments used tetrahydrofuran as the solvent, 1.0 MPa H_2_, reaction temperature 180°C for 6 h. Table 2 compares the performance of the different catalysts. The conversion rate of HMF and the selectivity to DMF on the CoAl-MMO catalyst were both very low, with the reaction producing mainly intermediate products. CoNiAl-MMO and CuCoAl-MMO were prepared by introducing Ni and Cu elements into the catalyst, respectively. The conversion rate of HMF was improved, and the selectivity of DMF was also significantly improved. Combining the four metals, the CuCoNiAl-MMO catalyst offered outstanding performance, with a HMF conversion of 99.8% and the selectivity of DMF is 95.3%. Therefore, CuCoNiAl-MMO is an excellent catalyst for this hydrogenation reaction.

**TABLE 2:** Performance comparison of different catalysts for the selective hydrogenolysis of HMF.

Catalyst	Conversion (%)	Selectivity (%)		
		DMF	5-MF	5-MFA
CoAl-MMO	15.9	44.7	41.5	13.8
CoNiAl-MMO	31.4	84.5	11.6	3.9
CuCoAl-MMO	39.5	81.6	13.9	4.5
CuCoNiAl-MMO	99.8	95.3	2.2	0.4

Reaction conditions: HMF (0.25 g), tetrahydrofuran (THF, 10 ml), catalyst (0.1 g), *T* = 180°C, *p*(H_2_) = 1.0 MPa, 6 h.

#### 3.2.2 Exploration of Experimental Conditions

**FIGURE 9:**
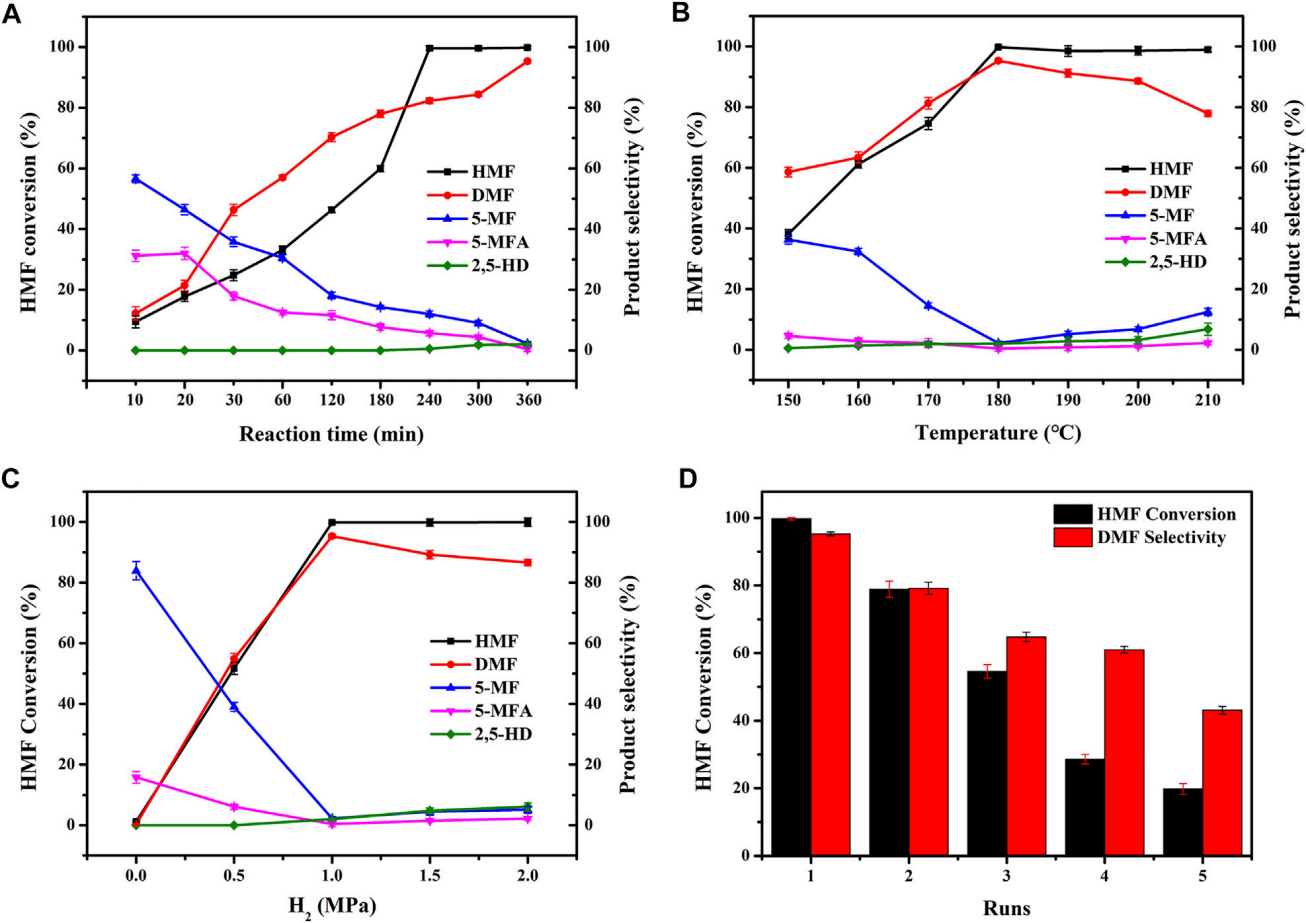
Effect of **(A)** reaction time, **(B)** temperature, **(C)** H_2_ pressure, and **(D)** catalytic test runs on the HMF hydrogenation performance of CuCoNiAl-MMO. Reaction conditions: **(A)** 0.1 g catalyst, 1.0 MPa H_2_, 0.25 g HMF, 180°C **(B)** 0.1 g catalyst, 1.0 MPa H_2_, 0.25 g HMF, 6 h **(C)** 0.1 g catalyst, 0.25 g HMF, 6 h, 180°C, and **(D)** 0.1 g catalyst, 1.0 MPa H_2_, 0.25 g HMF, 6 h, 180°C.

In order to further investigate the excellent catalytic performance of CuCoNiAl-MMO for the selective hydrogenation of HMF to DMF, we explored the effects of reaction time, temperature and H_2_ pressure on the reaction (Figures 9A–C). Figure 9A shows the effect of reaction time. We analyzed the reaction mixture at reaction times of from 10 min to 6 h. The conversion of HMF and the selectivity to DMF increased gradually over the first 4 h, with the HMF conversion plateauing from 4–6 h. At 6 h, the HMF conversion and selectivity to DMF were 99.8 and 95.3%, respectively. Intermediates such as 5-MF, 5-MFA decreased with reaction times, whereas the by-product 2,5-hexanedione (2,5-HD) formed at the longer reaction times. Figure 9B shows the effect of reaction temperature on HMF hydrogenation over the CuCoNiAl-MMO catalyst. In the temperature range of 150–180°C, the conversion rate of HMF and the selectivity to DMF gradually increased with the increase of temperature, whilst the content of 5-MF and 5-MFA progressively decreased. The reaction was optimal around 180°C. Above 180°C, the content of the intermediate products (5-MF) and by-products (2,5-HD) increased, since DMF can be hydrogenated to generate 2,5-HD. Figure 9C shows the influence of hydrogen pressure on HMF hydrogenation over the CuCoNiAl-MMO catalyst. When no hydrogen was in the reaction system, the conversion rate of HMF is almost negligible. The conversion rate of HMF and the selectivity of DMF both increased with an increase in the hydrogen pressure, with the best catalyst activity and selectivity to DMF being obtained at a hydrogen pressure is 1.0 MPa. When the hydrogen pressure increased further, DMF was hydrogenated to 2,5-HD, the lowering the selectivity to DMF. Therefore, considering the safety of the experiment, we chose hydrogen pressure of 1.0 MPa for subsequent catalytic tests. Based on the results, we could deduce the reaction path of HMF conversion into DMF. HMF hydrogenolysis dehydration produces 5-MF, with the 5-MF then hydrogenated to produce 5-MFA. Finally, 5-MFA is further hydrogenolyzed into DMF. In addition, we also tested the recycling performance of the catalyst (Figure 9D). After three cycles use of the catalyst, the catalytic performance dropped significantly. The reasons for the deactivation of the catalyst are discussed below.

#### 3.2.3 ICP-AES

In order to explore the reason why the catalytic performance of CuCoNiAl-MMO catalyst decreased sharply after three cycles of HMF hydrogenation tests, ICP-AES was used to probe the bulk chemical composition of CuCoNiAl-MMO and CuCoNiAl-MMO-3, with the aim of identifying any element losses during the catalyst tests. As shown in Table 3, the concentrations of Co and Al decreased significantly after three cycles of catalyst tests. Among them, Co is an important active metal, and its reduction affects the catalytic performance of the catalyst. The Al-containing component as a carrier has the effect of inhibiting the aggregation of metal particles and ensuring high dispersibility, and its reduction reduces the stability of the catalyst structure, thereby affecting the performance of the catalyst.

**TABLE 3:** The concentration of Cu, Co, Ni, Al in CuCoNiAl-MMO and CuCoNiAl-MMO-3 catalysts.

Catalyst	Cu (%)	Co (%)	Ni (%)	Al (%)
CuCoNiAl-MMO	14.00	23.59	41.84	20.56
CuCoNiAl-MMO-3	16.16	18.93	49.40	15.50

**FIGURE 10:**
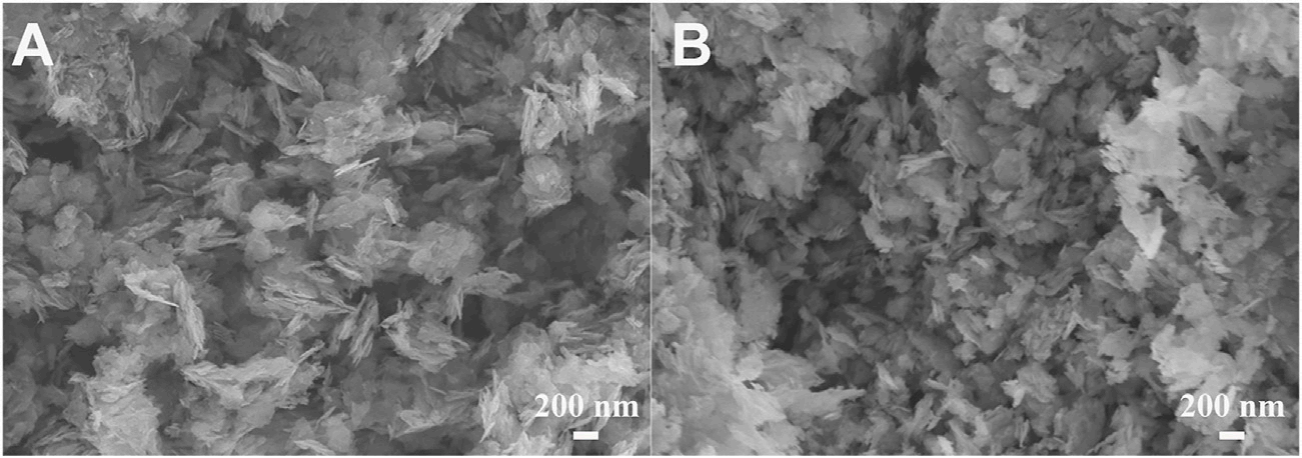
SEM images of **(A)** CuCoNiAl-MMO, **(B)** CuCoNiAl-MMO-3.

Figure 10 shows SEM images for the fresh and used CuCoNiAl-MMO catalyst. After the three test cycles, the resulting CuCoNiAl-MMO-3 catalyst had a denser structure with smaller sheet sizes. This might have reduced the availability of active sites on the catalyst surface, thereby reducing the hydrogenation performance. In addition, the CuCoNiAl-MMO-3 catalyst was also characterized by H_2_-TPR to study the changes caused by the catalytic tests. Figure 8 (curve d) shows that the reduction peaks moved to high temperature using the catalyst three times for HMF hydrogenation (i.e., the surface species were less reducible compared to the fresh catalyst). This was likely due to particle aggregation and a change in the type of metal/oxide species on catalyst surface.

#### 3.2.4 NH_3_-TPD

**FIGURE 11:**
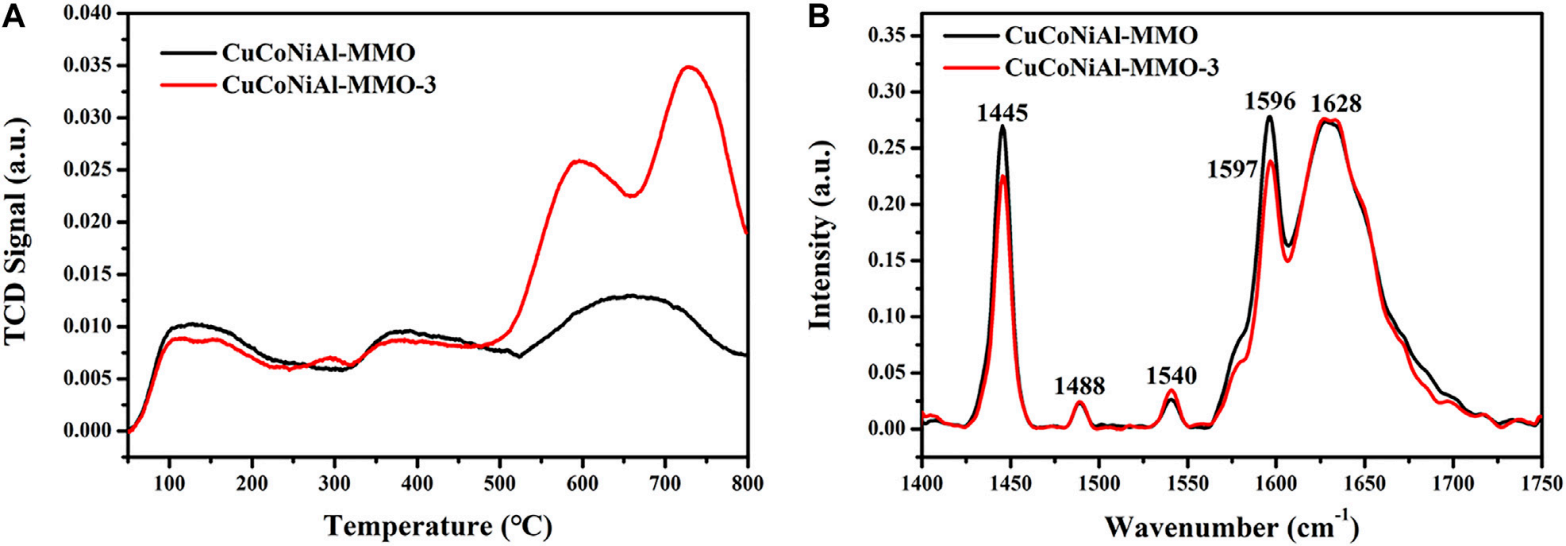
Characterization of surface acidity of the different catalysts. **(A)** NH_3_-TPD **(B)** Py-IR spectra for CuCoNiAl-MMO and CuCoNiAl-MMO-3.

NH_3_-TPD was used to examine the acid sites on the surface of CuCoNiAl-MMO and CuCoNiAl-MMO-3. Results are shown in Figure 11A. Based on the NH_3_ desorption temperature, the strengths of the acidic sites can be classified as weak (<250°C), medium (250–400 C), and strong (>400 C) (Srivastava et al., 2017). Both CuCoNiAl-MMO and CuCoNiAl-MMO-3 showed NH_3_ desorption peaks in the 100–200 C, 300–500 C, and 500–800 C regions, indicating that each catalyst contained weak acid, medium acid and strong acid sites. After three cycles of use, the amount of strong acid sites in the catalyst increased significantly. Strong acid sites are involved in C-C bond cleavage reactions such as catalytic cracking, skeletal isomerization, transalkylation, and disproportionation. Weak acid centers are involved in reactions of C-H bond cleavage, such as hydrogen transfer, hydration, cyclization, alkylation, etc. However, if the catalyst is too acidic, it will be deactivated by carbon deposition. Therefore, the abundance of strong acid sites is not conducive for HMF hydrogenolysis to DMF. Next, pyridine adsorption infrared tests (Py-IR) were conducted. As shown in Figure 11B, it can be seen that CuCoNiAl-MMO has more acidic sites for adsorption than CuCoNiAl-MMO-3. Both catalysts showed bands at 1,445 cm^−1^ and 1,488 cm^−1^, consistent with the adsorption of pyridine on Lewis acid sites. In addition, a weak absorption peak at 1,540 cm^−1^ was seen due to pyridine adsorption on Brønsted acid (B) sites. The adsorption band at 1,596 cm^−1^ arises from hydrogen bonding between pyridine and OH (Brønsted acid center) (Zhao and Lercher, 2012). Notably, the amount of Lewis acid and Brønsted acid sites determined by Py-IR on the catalysts were much lower compared with those identified by NH_3_-TPD analysis, consistent with the presence of a large quantity of acidic OH groups on the surface of the catalysts (Zhang and Chen, 2017). In the presence of acid groups on the catalyst surface, the hydroxyl group (-CH_2_-OH) in HMF preferentially undergoes a dehydroxylation reaction to form 5-MF (Guo et al., 2020).

### 3.3 Experimental Principle Exploration

**FIGURE 12:**
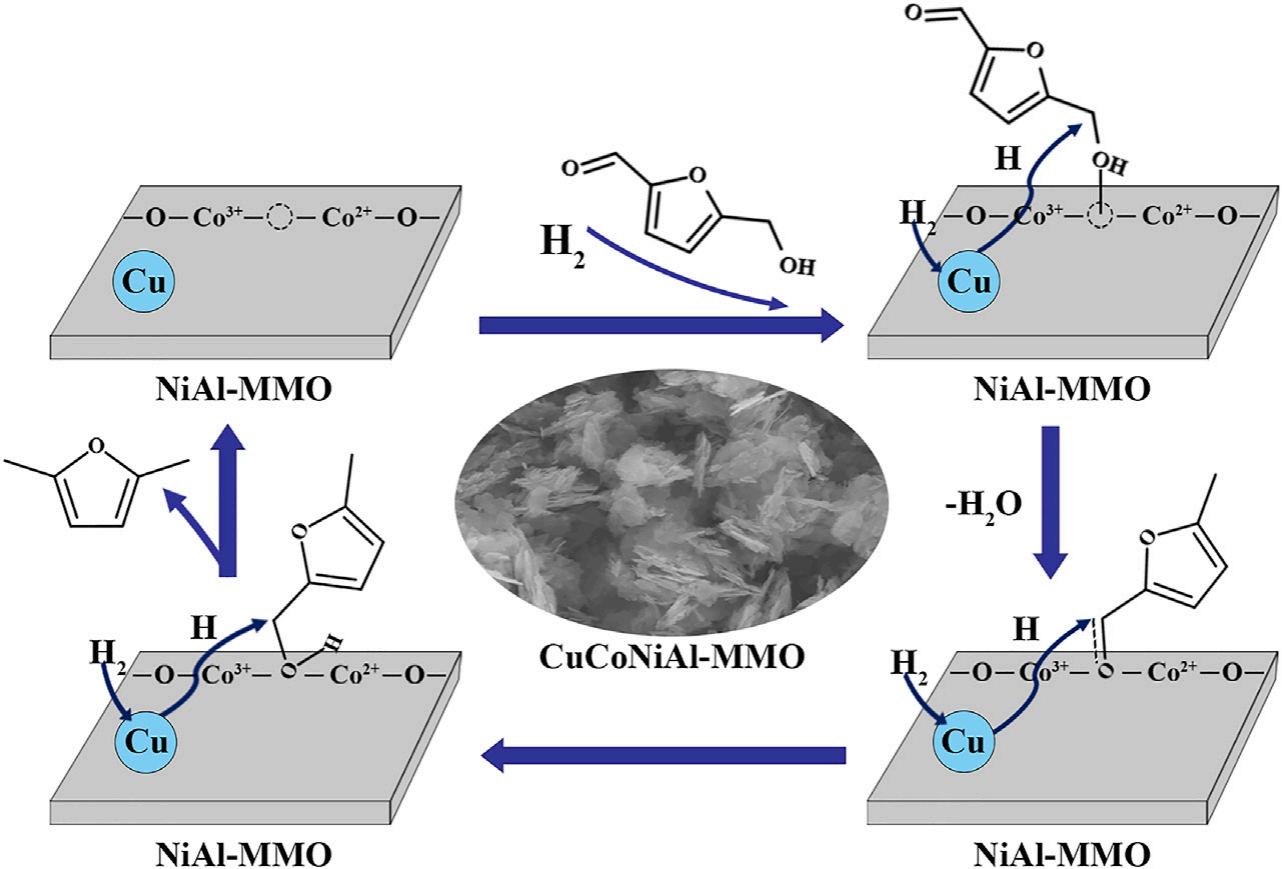
Reaction mechanism for DMF formation by HMF hydrogenolysis on CuCoNiAl-MMO.

Figure 12 illustrates a possible reaction mechanism for the hydrogenation of HMF on CuCoNiAl-MMO. There are two general ways to hydrogenate HMF to produce DMF (Figure 1). Route 1: The aldehyde group on HMF is hydrogenated to generate DHMF, after which the -OH on DHMF is hydrogenated and dehydrated to form 5-MFA, and finally, the -OH on 5-MFA is then hydrogenated and dehydrated to form the final product DMF. Route 2: Hydrogenation and dehydration of -OH on HMF to produce MF, hydrogenation of the aldehyde group on 5-MF to produce 5-MFA, and -OH on 5-MFA hydrogenated and dehydrated to produce the final product DMF. Both approaches have a common intermediate product 5-MFA. Our experiments identified 5-MF is the intermediate product with no DHMF being detected, thus the CuCoNiAl-MMO catalyst uses route 2: HMF→5-MF→5-MFA→DMF. As shown in Figure 12: First, the metal nanoparticles (Cu) and Lewis acid sites (CoO_x_) on the catalyst surface can activate H_2_ and -CH_2_OH, respectively. The oxygen in hydroxymethylfurfural -CH_2_-OH is activated by Lewis acid sites (CoO_x_) and then attacked by H atoms created by the metal nanoparticles (Cu), leading to hydrodeoxygenation to 5-MF. Secondly, the aldehyde groups in 5-MF were activated by the oxygen vacancies formed by Co^2+^, and hydrogenated to form 5-MFA. Finally, the -OH hydrodehydration on 5-MFA produces DMF, which is the final product. As a carrier, NiAl-MMO can isolate metal crystallites, inhibit sintering and increase the specific surface area of the catalyst. In addition to the main reaction pathways, 2-hexanol and 2,5-HD are formed as by-products from DMF hydrogenation.

## 4 Conclusion

A CuCoNiAl-MMO catalyst was successively prepared by calcination a CuCoNiAl-LDH precursor at 500°C for 5 h in N_2_. The developed CuCoNiAl-MMO catalyst offered excellent initial atctivity and selectivity for the hydrogenolysis of HMF to DMF under very mild conditions (tetrahydrofuran solvent, 180°C for 6 h, 1.0 MPa H_2_). Under these conditions, CuCoNiAl-MMO afforded a HMF conversion rate of 99.8% and the DMF selectivity of 95.3%. Loss of Co and Al during the catalytic tests, together with densification of the catalyst structure and an increase in the amount of strong acid sites, meant that the initial catalytic activity was lost after three cycles of catalytic tests. LDH precursors offer a facile route to prepare highly active catalysts for HMF hydrogenation to DMF under mild conditions, though further work is needed to stabilize the structure/activity of the initially formed MMO-based catalysts.

## Data Availability

The original contributions presented in the study are included in the article/Supplementary Material, further inquiries can be directed to the corresponding author.
